# Nonphosphorylatable PEA15 mutant inhibits epithelial-mesenchymal transition in triple-negative breast cancer partly through the regulation of IL-8 expression

**DOI:** 10.1007/s10549-021-06316-2

**Published:** 2021-07-09

**Authors:** Jihyun Park, Moises J. Tacam, Gaurav Chauhan, Evan N. Cohen, Maria Gagliardi, Lakesla R. Iles, Naoto T. Ueno, Venkata L. Battula, Yoo-Kyoung Sohn, Xiaoping Wang, Hak-Sung Kim, Savitri Krishnamurthy, Natalie W. Fowlkes, Morgan M. Green, Geoffrey A. Bartholomeusz, Debu Tripathy, James M. Reuben, Chandra Bartholomeusz

**Affiliations:** 1 Section of Translational Breast Cancer Research, Department of Breast Medical Oncology, The University of Texas MD Anderson Cancer Center, Houston, TX USA; 2 Department of Hematopathology, The University of Texas MD Anderson Cancer Center, Houston, TX USA; 3 Department of Leukemia, The University of Texas MD Anderson Cancer Center, Houston, TX USA; 4 Department of Experimental Therapeutics, The University of Texas MD Anderson Cancer Center, Houston, TX USA; 5 Department of Biological Sciences, Korea Advanced Institute of Science and Technology, Daejeon, Korea; 6 Department of Pathology, The University of Texas MD Anderson Cancer Center, Houston, TX USA; 7 Department of Veterinary Medicine & Surgery, The University of Texas MD Anderson Cancer Center, Houston, TX USA; 8 Department of Breast Medical Oncology, The University of Texas MD Anderson Cancer Center, 1515 Holcombe Boulevard, Houston, TX 77030 USA

**Keywords:** PEA15, EMT, Triple-negative breast cancer, IL-8, Ets-1

## Abstract

**Background:**

Triple-negative breast cancer (TNBC) is an aggressive breast cancer subtype that lacks targeted therapies. Patients with TNBC have a very poor prognosis because the disease often metastasizes. New treatment approaches addressing drivers of metastasis and tumor growth are crucial to improving patient outcomes. Developing targeted gene therapy is thus a high priority for TNBC patients. PEA15 (phosphoprotein enriched in astrocytes, 15 kDa) is known to bind to ERK, preventing ERK from being translocated to the nucleus and hence blocking its activity. The biological function of PEA15 is tightly regulated by its phosphorylation at Ser104 and Ser116. However, the function and impact of phosphorylation status of PEA15 in the regulation of TNBC metastasis and in epithelial-to-mesenchymal transition (EMT) are not well understood.

**Methods:**

We established stable cell lines overexpressing nonphosphorylatable (PEA15-AA) and phospho-mimetic (PEA15-DD) mutants. To dissect specific cellular mechanisms regulated by PEA15 phosphorylation status, we performed RT-PCR immune and metastasis arrays. In vivo mouse models were used to determine the effects of PEA15 phosphorylation on tumor growth and metastasis.

**Results:**

We found that the nonphosphorylatable mutant PEA15-AA prevented formation of mammospheres and expression of EMT markers in vitro and decreased tumor growth and lung metastasis in in vivo experiments when compared to control, PEA15-WT and phosphomimetic PEA15-DD. However, phosphomimetic mutant PEA15-DD promoted migration, mesenchymal marker expression, tumorigenesis, and lung metastasis in the mouse model. PEA15-AA-mediated inhibition of breast cancer cell migratory capacity and tumorigenesis was the partial result of decreased expression of interleukin-8 (IL-8). Further, we identified that expression of IL-8 was possibly mediated through one of the ERK downstream molecules, Ets-1.

**Conclusions:**

Our results show that PEA15 phosphorylation status serves as an important regulator for PEA15’s dual role as an oncogene or tumor suppressor and support the potential of PEA15-AA as a therapeutic strategy for treatment of TNBC.

**Supplementary Information:**

The online version contains supplementary material available at 10.1007/s10549-021-06316-2.

## Introduction

Among American women, breast cancer is the most common cancer diagnosed and the second leading cause of cancer death [1–3]. Triple-negative breast cancer (TNBC) is an aggressive subtype of breast cancer characterized by poor prognosis. TNBC is highly proliferative and sensitive to systemic chemotherapies. However, despite its relative chemosensitivity, the 5-year overall survival rate for patients with TNBC is only 60–70% [4]. The cause of death in many patients with TNBC is recurrence, which presents commonly as metastasis. To reduce metastasis, developing additional treatment strategies, such as targeted gene therapy, for TNBC patients is a high priority.

We are investigating as a potential target PEA15 (phosphoprotein enriched in astrocytes), a 15-kDa phosphoprotein that is ubiquitously expressed in breast cancer. PEA15 overexpression significantly inhibited tumor growth and suppressed DNA synthesis in a TNBC xenograft model [5]. Others have shown that PEA15 overexpression inhibited invasion by binding to ERK, and decreased PEA15 expression levels were observed in metastatic breast cancer cells, suggesting that PEA15 may be a suppressor of metastasis [6–8].

In the current study, we investigated the function and impact of phosphorylation status of PEA15 in the regulation of TNBC metastasis and in epithelial-to-mesenchymal transition (EMT), and we further examined the mechanisms involved. The PEA15 protein contains two major serine residues, Ser104 and Ser116, at the C-terminus that are phosphorylatable [9, 10]. We previously found that a nonphosphorylatable mutant of PEA15 inhibited ovarian cancer cell proliferation and tumor growth through partial inhibition of the β-catenin signaling pathway [11]. EMT is characterized by loss of epithelial cell junction proteins and gain of mesenchymal markers [12, 13]. It has been proposed that EMT-like processes allows primary tumor cells to disassemble and migrate to distant tissue or organ sites [12, 13]. EMT has been suggested to be a reason for the aggressiveness of basal-like (BL) breast cancer [14]. In the present study, using phosphoinhibitory and phosphomimetic PEA15 mutants, we identified a critical role of PEA15 phosphorylation in regulating EMT and metastasis in TNBC cells. We observed that nonphosphorylatable PEA15-AA strongly suppressed migration in vitro. The results seen were partially dependent on IL-8, which is regulated by ERK-responsive transcription factor Ets-1. We further found that nonphosphorylatable PEA15-AA inhibited formation of mammospheres and showed a decrease in mesenchymal markers in vitro and decreased tumor growth and lung metastasis in vivo when compared to phosphomimetic PEA15-DD. Taken together, our results present novel insight into the role of phosphorylation status of PEA15 in TNBC and suggest nonphosphorylatable PEA15-AA as a promising treatment strategy for TNBC.

## Materials and methods

### Cell lines and culture conditions

The human breast adenocarcinoma cell lines MDA-MB-231 and MDA-MB-468 were obtained from the American Type Culture Collection. MDA-MB-231 and MDA-MB-468 cells were grown in DMEM/F12 (Life Technologies) supplemented with 10% FBS and penicillin/streptomycin and maintained in a humidified incubator at 37 °C containing 5% CO_2_. CRISPR-edited PEA-15-KO clones in MDA-MB-231 were established.

### Western blot analysis

Cells were washed with PBS (pH 7.4) and then lysed in lysis buffer [20 mmol/L Na_2_PO_4_ (pH 7.4), 150 mmol/L NaCl, 1% Triton X-100, 1% aprotinin, 1 mmol/L phenylmethylsulfonyl fluoride, 100 mmol/L NaF, and 2 mmol/L Na_3_VO_4_] as previously described [5]. Further, western blot analysis was performed as described in Supplementary methods.

### Transfection

As described previously [11], MDA-MB-468 and MDA-MB-231cells were transfected with expression plasmids containing empty vector (pcDNA3-HA), PEA15-AA (pcDNA3-HA-PEA15-AA), or PEA15-DD (pcDNA3-HA-PEA15-DD) using FuGENE transfection reagent according to the manufacturer’s instructions. These constructs were kindly provided by Dr. Mark H. Ginsberg (University of California San Diego, La Jolla, CA).

### Colony formation and Mammosphere formation assays

Colony formation and Mammosphere formation assays were performed as described in Supplementary methods.

### Transwell migration assay

As described previously [15], migration assay were performed in triplicate using a 24-well micro-chemotaxis chamber. The assays were further conducted as described in Supplementary methods.

### In vivo tumorigenicity assays

Four-to six-week-old female NOD/SCID mice were used to establish MDA-MB-468 breast cancer xenografts stably expressing empty vector or the PEA15 mutants. Cells were prepared in a 1:1 mixture of PBS and growth factor-reduced Matrigel (BD Biosciences) at 4 × 10^6^ cells in 100 µL, and this cell suspension was injected into the mammary fat pads of mice.

MDA-MB-468 cells (5 × 10^6^ cells in 100 μL Matrigel) were inoculated into the mammary fat pads of NOD/SCID mice. When tumor size reached approximately 200 mm^3^, either Rb-PEA15-AA or Rb-control was intravenously injected into the mice twice a week for 6 weeks. The mice were treated with equimolar amounts of each protein (27 μM/dose).

Tumor volume (mm^3^) was calculated, and changes in tumor volumes were tested for statistical significance with the Mann–Whitney test or Student's two-tailed *t* test.

### In vivo experimental metastasis assay

MDA-MB-231 PEA15-KO cells were used to establish stable cell lines overexpressing empty vector, PEA15-WT, PEA15-AA and PEA15-DD. Female athymic *nu/nu* mice, age 6 weeks, were purchased (Envigo). MDA-MB-231 PEA15-KO cells stably expressing PEA15 constructs (2 × 10^6^ cells/100 µL PBS) were injected into the tail veins of the mice. At 8 weeks after inoculation, animals were euthanized, and the lung tissues were analyzed for metastasis. The experimental metastasis model will allow us to determine the two late events of the metastatic process, extravasation and organ colonization.

### Immunohistochemistry

As described previously [15], immunohistochemistry was performed as further described in Supplementary methods.

### RT^2^ profiler PCR array

RT^2^ Profiler Human Cancer Inflammation and Immunity Crosstalk PCR Array (Qiagen) was performed using the Bio-Rad CFX96 cycler [16]. The expression levels were quantified relative to the values obtained for housekeeping genes (*ACTB, B2M, GAPDH, HPRT1*, and *RPLP0*). Data were analyzed using Qiagen web-based software (https://www.qiagen.com/us/shop/genes-and-pathways/data-analysis-center-overview-page/).

### Statistical analysis

Data are given as mean ± SD. Student’s *t* test or ANOVA was performed to compare the differences between two or more than two groups. *P* < 0.05 was considered a statistically significant value.

## Results

### Phosphoinhibitory PEA15 (PEA15-AA) significantly reduces colony formation and the cancer stem cell phenotype in vitro

MDA-MB-468 cells were selected because these cells are known to be tumorigenic in a xenograft model [5] and they demonstrated low endogenous expression of PEA15. To further study the effect of the phosphorylation status of PEA15 on metastasis in vivo, we selected a highly metastatic TNBC cell line, MDA-MB-231, even though it has high expression of PEA15 (Figure S1). To circumvent the effect of endogenous PEA15, the endogenous PEA15 was knocked out in the MDA-MB-231 cells (MDA-MB-231 PEA15-KO) using CRISPR/Cas9. We either transiently transfected or established stable cell lines overexpressing empty vector (PEA15-V), wild-type PEA15 (PEA15-WT), nonphosphorylatable mutant (PEA15-AA), and phosphomimetic mutant (PEA15-DD).

**Fig. 1:**
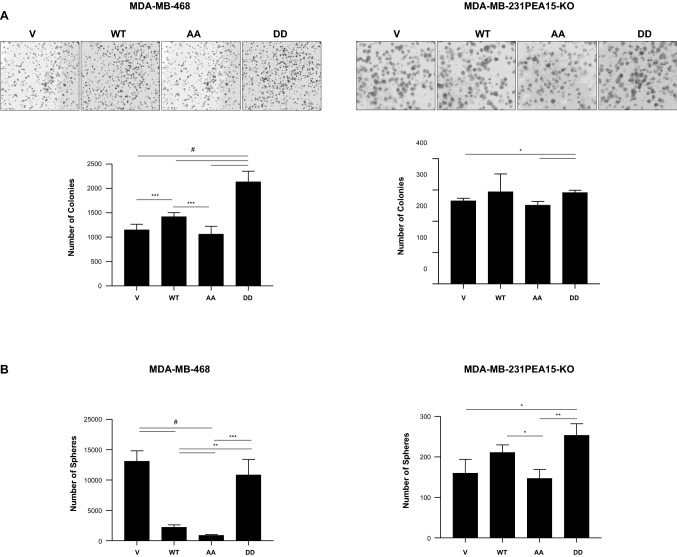
Effect of PEA15-AA on colony formation and the CSC phenotype in TNBC cells. In vitro **A**, colony formation assay and **B**, mammosphere formation assay were performed on MDA-MB-468 stable overexpressing clones and MDA-MB-231 PEA15-knockout transiently transfected cells and collected after 7–10 days. Results are representative of a minimum of two independent experiments. Error bars represent the mean (*n* = 6) ± SD. *P < 0.05, **P < 0.01, ***P < 0.001, and #*P* < 0.0001 by Welch-ANOVA with Games-Howell’s multiple comparisons test

We tested the effects of PEA15 phosphorylation status on cell proliferation and observed no significant difference between cells expressing PEA15-AA and PEA15-DD compared to the vector control cells (Figure S2). Next, we assessed the effect of PEA15 phosphorylation on colony formation ability in TNBC cells. PEA15-AA-expressing cells formed fewer colonies than did the PEA15-WT-expressing or PEA15-DD-expressing MDA-MB-468 and MDA-MB-231-PEA15-KO cells (Fig. 1A). The EMT phenotype is strongly correlated with the ability of cells to form mammospheres, which are an indicator of self-renewal and cancer stem cell (CSC) properties [17]. To determine the role of PEA15 phosphorylation on the CSC-like phenotype, we performed a mammosphere formation assay. As shown in Fig. 1B, PEA15-AA significantly decreased the formation of mammospheres compared to PEA15-WT-expressing or PEA15-DD-expressing cells. Taken together, these results strongly suggest that the unphosphorylated PEA15-AA contributed to the suppression of colony formation and stemness phenotype in TNBC cells.

### PEA15-AA suppresses cell motility and EMT in vitro

**Fig. 2:**
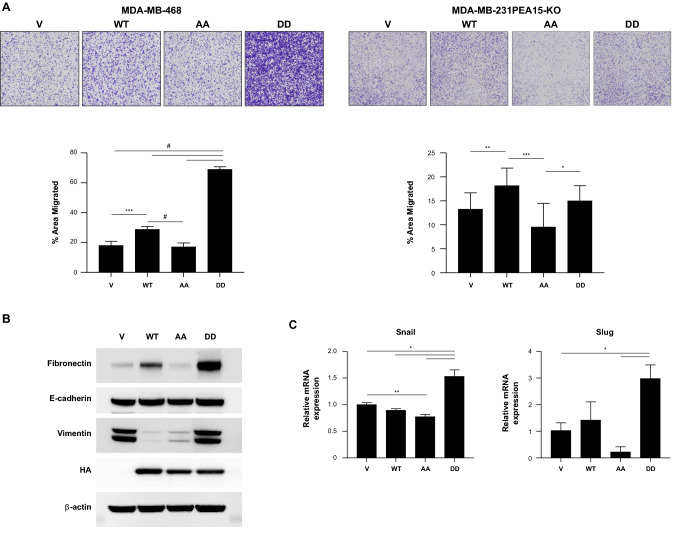
Effect of PEA15-AA on migration and EMT in vitro*.* **A**, Cell migration was investigated using transwell chambers for MDA-MB-468 stable overexpressing clones and MDA-MB-231 PEA15-KO transiently transfected cells. After staining, ImageJ software was used to quantify the percent area migrated through six sections of each membrane. Images were taken at 20× magnifications. **B**, Immunoblotting of MDA-MB-468 stable cells for EMT markers (fibronectin, E-cadherin and vimentin), HA-tag, and loading control β-actin. RT-qPCR was performed for EMT transcriptional factors (Snail and Slug) and GAPDH loading control. Relative expressions were normalized to empty vector-endogenous MDA-MB-468. Results are representative of a minimum of two independent experiments. Error bars represent the mean (**A**, *n* = 6; **B**, *n* = 3) ± SD. *P < 0.05, **P < 0.01, ***P < 0.001, and #*P* < 0.0001 by Welch-ANOVA with Games-Howell’s multiple comparisons test

To study the role of phosphorylation of PEA15 in the TNBC cell migratory phenotype, we performed an in vitro migration assay. Compared with control PEA15-WT and PEA15-DD cells, PEA15-AA cells had effective suppression of migration (Fig. 2A). To assess the effect of phosphorylation status of PEA15 on EMT, the protein levels of EMT markers were analyzed. Overexpression of PEA15-AA in MDA-MB-468 cells decreased the expression of fibronectin and vimentin (a mesenchymal markers) (Fig. 2B). Furthermore, the mRNA levels of EMT-inducing transcription factors, Snail and Slug were significantly decreased in PEA15-AA-expressing cells (Fig. 2C). In contrast, we observed that phosphomimetic PEA15-DD-expressing cells had increased expression of fibronectin and vimentin (Fig. 2B) and Snail and Slug (Fig. 2C). Together, these results indicate that unphosphorylated PEA15 suppresses cell motility and EMT of TNBC cells in vitro*.* These results suggest that one potential pathway by which PEA15 phosphorylation status regulates breast cancer cell migration is through upregulating or downregulating EMT-related molecules.

### PEA15-AA inhibits tumor growth and lung metastasis compared to PEA15-DD in a breast cancer mouse model

**Fig. 3:**
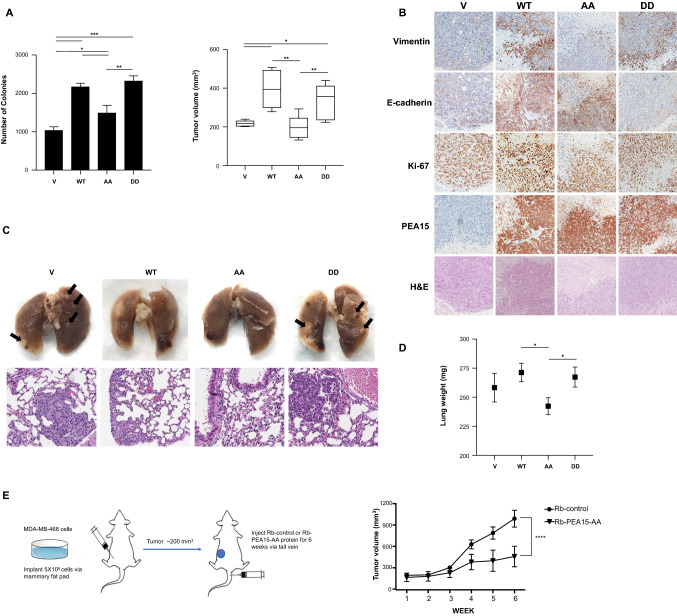
Effect of PEA15-AA on anchorage-independent growth, tumor growth, EMT, and metastasis in vivo. **A**, Anchorage-independent growth in soft agar for MDA-MB-468 stable overexpressing clones, used as a predictive in vitro marker of tumorgenicity. Representative results for two independent experiments. Error bars represent the mean (*n* = 3) ± SD. *P < 0.05, **P < 0.01, and ***P < 0.001 by Welch-ANOVA with Games-Howell’s multiple comparisons test. The tumorigenic effect of PEA15 phosphorylation was evaluated by injecting empty Vector, WT, AA, and DD stable clones (MDA-MB-468 cells) into the mammary fat pads of NOD/SCID mice. Tumor volumes were analyzed at 8 weeks and represented as box-and-whisker plots (5–95% confidence intervals) to show the data structure. **B**, Immunohistochemical staining showing expression levels of vimentin, E-cadherin, Ki-67, and HA-tag (HA-PEA-15). Images were taken at 20× magnifications. H&E, hematoxylin–eosin **C**, The effect of PEA15 phosphorylation status on metastasis was assessed by injecting MDA-MB-231 PEA15-knockout clones containing empty Vector, WT, AA, and DD into the tail veins of nude mice. Representative photographs of fixed lungs and H&E-stained images of lung sections. Images were taken at 20× magnifications. Arrows indicate metastatic lesions. **D**, Lungs were weighed to quantify metastasis and error bars represent the mean ± SEM. For the box-and-whisker plot in **A** and for graph **D**, unpaired *t* test with Welch’s corrections was used. *P < 0.05, and **P < 0.01. **E**, Following inoculation with MDA-MB-468 cells, tumor volumes were measured in xenograft mice weekly after treatment with Rb-control or Rb-PEA15-AA protein. No significant difference in body weight was seen over the course of the experiment (not shown). Data represent the mean (*n* = 10) ± SEM. ****P < 0.0001

We first assessed the anchorage-independent growth of the MDA-MB-468 stable overexpressing clones in soft agar as an indirect test of their tumorigenicity. We observed that the PEA15-AA stable transfectants formed fewer colonies (i.e., were less tumorigenic) than did the PEA15-WT- or PEA15-DD-transfected cells (Fig. 3A, left panel).

To identify the most tumor-suppressive form of PEA15, we examined the effects of PEA15-AA- and PEA15-DD-overexpressing stable MDA-MB-468 cells in vivo by injecting them into the mammary fat pads of NOD/SCID mice. At 8 weeks, the tumors in the mice injected with PEA15-AA were significantly smaller when compared with mice that were injected with PEA15-WT and PEA15-DD. Animals were euthanized at 8 weeks, and the expression levels of EMT markers in the tumors were analyzed with immunohistochemical staining. As expected, expression of mesenchymal marker vimentin was inhibited in PEA15-AA-expressing tumors compared with PEA15-WT or PEA15-DD-expressing tumors (Fig. 3B). The level of epithelial marker E-cadherin was decreased in PEA15-DD-expressing tumors. However, PEA15 phosphorylation status did not affect the expression of proliferation marker Ki-67. Next, we assessed the impact of PEA15 phosphorylation on lung metastasis. Vector, PEA-15-WT-, PEA15-AA-, and PEA15-DD-overexpressing stable MDA-MB-231 PEA15-KO cells were injected into the tail veins of nude mice. At 8 weeks after inoculation, mice were euthanized and analyzed for lung metastasis burden. Figure 3C shows representative lung images that display the metastasis burden of animals injected with vector control, PEA15-WT-expressing, PEA15-AA-expressing, and PEA15-DD-expressing cells. Mice injected with PEA15-AA cells showed much less macroscopic metastasis/colonization in the lungs compared with the vector control and PEA15-DD-expressing groups; presence of tumor was confirmed histologically in group DD after H&E staining of formalin-fixed paraffin lung tissue sections. In addition, as shown in Fig. 3D, the weight of lungs from mice injected with PEA15-AA-expressing cells was lower than those from mice injected with PEA15-WT- or PEA15-DD-expressing cells. These results suggest that phosphorylation of PEA15 may stimulate tumor growth by modulating EMT-related molecules and further enhance metastasis of cancer cells. Additionally, we tested the antitumor efficacy of purified PEA15-AA protein combined with an EGFR-specific repebody (rEgH9) and a translocation domain (TDP) in vivo. The delivery platform is composed of a targeting moiety, translocation domain, and PEA15 derivative. The successfully developed rEgH9 and TDP were utilized by Ryou et al. [18] as an effective tool for cytosolic delivery of cargo proteins in an EGFR-specific manner, implying broad uses for cancer therapy. As seen in Fig. 3E, mice implanted with high-EGFR-expressing MDA-MB-468 cells showed significant inhibition of tumor growth when treated with Rb-PEA15-AA protein compared with Rb-control. Taken together, our data demonstrate that unphosphorylated PEA15-AA possesses an antitumor effect in vivo and thus offers a potential therapeutic agent for the treatment of breast cancer.

### PEA15 phosphorylation state regulates IL-8 expression

**Fig. 4:**
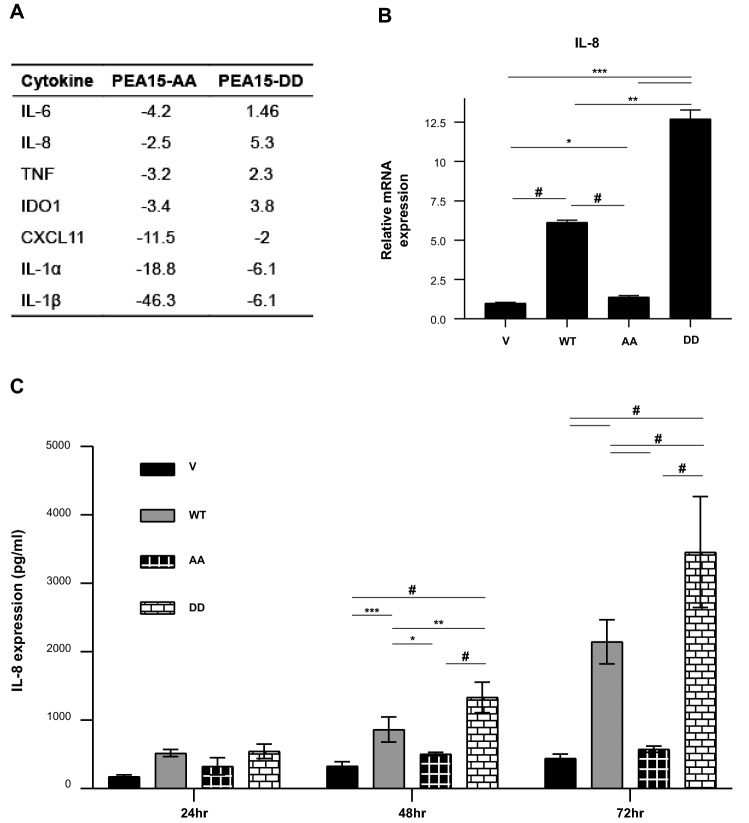
Determine if IL-8 is a potential downstream target of PEA15. **A**, RT^2^ Profiler Human Cancer Inflammation and Immunity Crosstalk PCR Array (Qiagen) revealed differentially expressed genes between PEA15-AA-expressing and PEA15-DD-expressing cells. **B**, RT-qPCR was performed for IL-8 and GAPDH as loading control. Relative expressions were normalized to empty vector in MDA-MB-468. Results are representative of two independent experiments (*n* = 3). Error bars represent mean ± SD. **C**, Secreted IL-8 levels were measured using human IL-8/CXCL8 Quantikine ELISA Kit (R&D Systems). MDA-MB-468 stable overexpressing clones were plated and conditional medium was collected at 24, 48, and 72 h. Results and errors bars represent mean ± SD for three independent collection experiments and were measured in duplicate wells (*n* = 6). *P < 0.05, **P < 0.01, and ***P < 0.001 by Welch-ANOVA with Games-Howell’s multiple comparisons test for **B** and **C**

We next studied the molecular mechanism by which nonphosphorylatable PEA15 inhibits cell migration and EMT. Our group [11] observed a strong antitumorigenic effect of unphosphorylated PEA15-AA in an ovarian cancer xenograft model even though both PEA15-AA and PEA15-DD exhibited a tumor-suppressive phenotype in vitro. Based on that study, we speculated that the tumor microenvironment is a critical factor in regulating tumor growth and progression. Thus, we performed the RT^2^ Profiler Human Cancer Inflammation and Immunity Crosstalk PCR Array to analyze the gene expression changes that occur as a result of phosphorylation status of PEA15. Interestingly, we found that IL-8 was one of the most downregulated genes in PEA15-AA-expressing cells and one of the most upregulated in PEA15-DD-expressing cells (Fig. 4A). As shown in Fig. 4B, we confirmed decreased expression of IL-8 mRNA in PEA15-AA-expressing cells. Further, IL-8 protein expression in these cells were consistently low at different timepoints (Fig. 4C). In addition, we observed an increased expression of IL-8 in PEA15-DD-expressing cells. IL-8 levels increased in PEA15-WT-expressing cells as well, however, this increase did not occur at the same rate as seen in PEA15-DD-expressing cells (Fig. 4C).

**Fig. 5:**
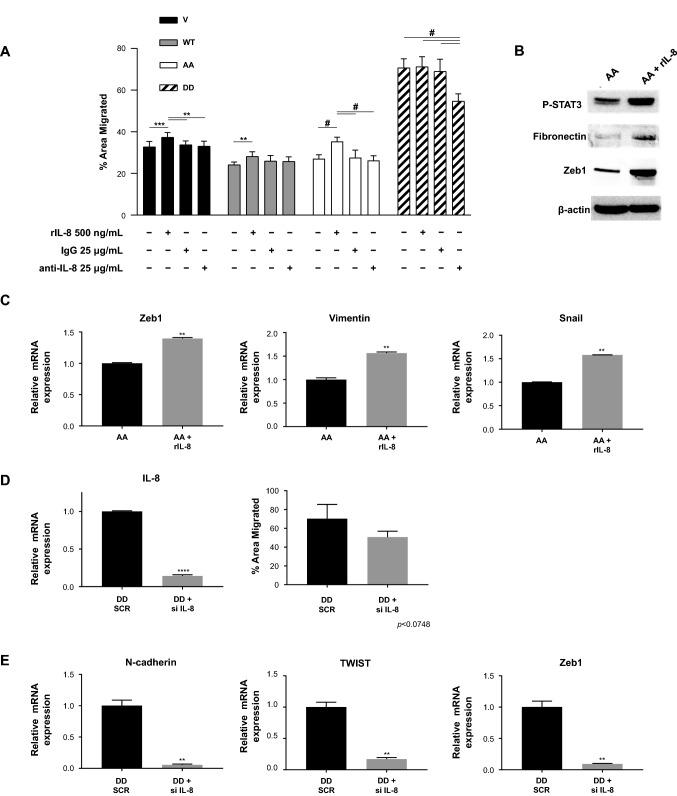
Assess if PEA15-AA’s inhibitory effect on TNBC migration capability is dependent on IL-8 downregulation. **A**, Migration capabilities were assessed using transwell chambers. Top chambers had serum-free media containing 1 × 10^6^ cells, and bottom chambers had either 0.5% FBS only, rIL-8 (500 ng/mL), IgG1 isotype control, or 25 μg/mL neutralizing IL-8 antibody (B&D Biosciences). Shown is one representation of two independent experiments. Error bars are mean (*n* = 6) ± SD. **P < 0.01, ***P < 0.001, and #*P* < 0.0001 by Welch-ANOVA with Games-Howell’s multiple comparisons test. **B**, Immunoblotting of MDA-MB-468 PEA15-AA-expressing clone following treatment with recombinant IL-8 for EMT markers (fibronectin and Zeb1), P-STAT3, and loading control β-actin, compared to non-treated control. **C**, RT-qPCR was performed for expression levels of EMT inducers (Zeb1, vimentin, and Snail) and GAPDH as loading control after rIL-8 treatment in the PEA15-AA-expressing cells. Relative expressions were normalized to the non-treated control group. **D**, IL-8 knockdown in the MDA-MB-468 PEA15-DD-expressing clone confirmed by RT-qPCR, and its effect on migration ability. **E**, RT-qPCR was performed for expression levels of EMT markers (N-cadherin, Twist, and Zeb1) and GAPDH loading control in an IL-8-knockdown PEA15-DD-expressing clone compared to the SCR siRNA control group. Results and errors bars represent mean (*n* = 4) ± SD. **P < 0.01, and #*P* < 0.0001 by unpaired *t* test with Welch’s corrections

IL-8 is also expressed by cancer cells undergoing EMT and shown to promote metastasis [17, 19–22]. The important role of IL-8 in promoting cell migration, and its reduced expression by PEA15-AA-expressing cells, led us to test whether decreased IL-8 expression in PEA15-AA cells was responsible for reduced migration. We tested whether recombinant IL-8 (rIL-8) could rescue the decreased migration of PEA15-AA-expressing MDA-MB-468 cells. We observed that recombinant IL-8 treatment rescued PEA15-AA-induced inhibition of migration (Fig. 5A). We observed that the PEA15-AA-expressing cells showed a more significant increase in migration (by 9%, *P* < 0.0001) compared to Vector and PEA-15-WT-expressing cells (Fig. 5A). No increase in migration was observed in PEA15-DD -expressing cells even in the presence of rIL8. Moreover, IL-8 treatment-induced expression of mesenchymal markers fibronectin, Zeb1, vimentin and snail in PEA15-AA-expressing cells (Fig. 5B,C). These results imply that IL-8 treatment is able to partly rescue the reduced migration caused by PEA15-AA.

Next, treating the cells with a neutralizing antibody against IL-8 (anti-IL-8) did not decrease migration in the empty Vector, PEA-15-WT or PEA-15-AA-expressing cells, but did decrease migration by 20% in PEA-15-DD-expressing cells compared to IgG isotype control. This confirmed that the high expression of IL-8 in the PEA-15-DD-expressing cells was partly responsible for its increased migratory capabilities.

To further confirm IL-8 as an important regulator in TNBC migration and EMT, we used IL-8 siRNA to knock down IL-8 expression in MDA-MB-468 cells stably expressing PEA15-DD. RT-qPCR analyses revealed that IL-8 siRNA successfully reduced the expression of IL-8 about 80% compared with control scrambled siRNA (Fig. 5D, left). We found that the migratory ability was partly decreased in PEA15-DD cells upon IL-8 siRNA treatment compared with control treatment (Fig. 5D, right). As expected, knockdown of IL-8 decreased the mesenchymal markers (n-cadherin, Twist, and Zeb1), as shown in Fig. 5E. These data demonstrate that IL-8 downregulation partially abolishes the increased cell migration and reverses the mesenchymal phenotype seen in the PEA15-DD-expressing cells and the effects of IL-8 expression could be altered by the phosphorylation status of PEA15.

### IL-8 downregulation by PEA15-AA is regulated via Ets-1

**Fig. 6:**
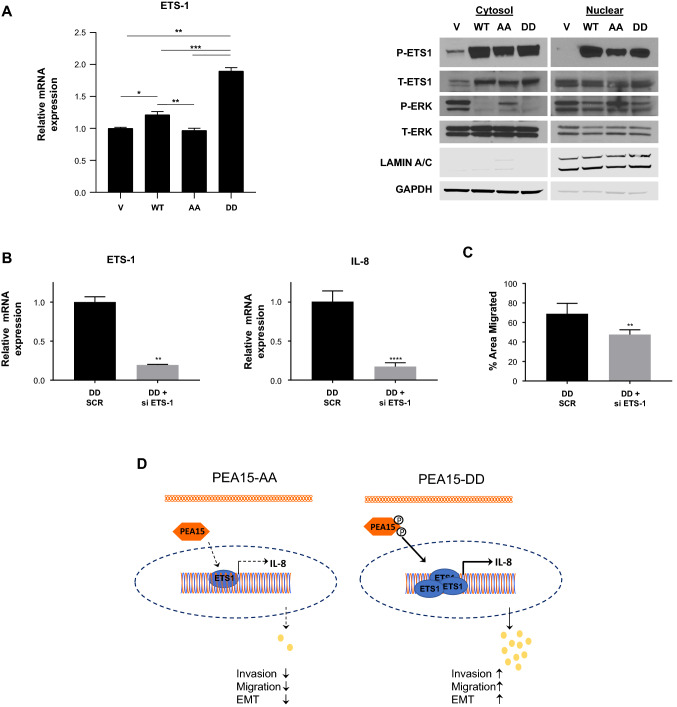
Determine if IL-8 downregulation by PEA15-AA is regulated via Ets-1. **A**, Relative mRNA expression level of Ets-1 was assessed by RT-qPCR in MDA-MB-468 stable overexpressing clones. Relative expressions were normalized to empty vector-endogenous MDA-MB-468. Results are representative of three independent experiments (*n* = 3). Cytoplasmic and nuclear extract were separately prepared and probed with phospho- and total ETS-1, phospho- and total ERK1/2, cytosolic loading control (GAPDH), and nuclear loading control (Lamin A/C). **B**, RT-qPCR analysis of Ets-1 to confirm Ets-1 knockdown in MDA-MB-468 PEA15-DD-overexpressing clone, and its impact on IL-8 mRNA expression compared to SCR siRNA control. **C**, Migration capabilities were investigated in Ets-1-knockdown MDA-MB-468 PEA15-DD-overexpressing clone compared to SCR siRNA control. **D**, Schematic diagram illustrating differing role between AA-overexpressing and DD-overexpressing clones. Error bars represent mean ± SD. *P < 0.05, **P < 0.01, and ***P < 0.001 using Welch-ANOVA with Games-Howell’s multiple comparisons test for **A**, and with unpaired *t* test with Welch’s corrections for **B** and **C**. **P < 0.01 and ****P < 0.0001

It has been reported that ERK downstream molecules cause tumor development and induce IL-8 expression in many cancer types. Furthermore, in neuroblastoma, IL-8 expression was upregulated in an Ets1-dependent manner, suggesting a critical link between Ets-1 and IL-8 in the angiogenesis and metastasis process [23]. Moreover, the IL-8 promoter contains binding sites for Ets-family transcription factors in hematopoietic cells [24]. These findings indicate that interruption of IL-8 signaling may be a potential targeted treatment in metastasis. To determine whether phosphorylation of PEA15 regulates IL-8 expression through ERK downstream signaling, we measured the mRNA levels of the ERK-responsive transcription factor Ets-1. We observed that the mRNA expression level of Ets-1 was significantly decreased in PEA15-AA-expressing cells compared with PEA15-WT- and PEA15-DD-expressing cells. On the other hand, Ets-1 expression was greatly increased in PEA15-DD-expressing cells (Fig. 6A, left).

Considering the possible functional relevance of Ets-1 cellular distribution, we further analyzed the levels of total Ets-1 and phosphorylated Ets-1 in fractionated cell lysates from MDA-MB-468 cells. Compared with PEA15-WT- and PEA15-DD-expressing cells, PEA15-AA-expressing cells showed reduction of phosphorylated Ets-1 in the nuclear fraction. In addition, we observed that the p-ERK level in the cytoplasm was higher in the PEA15-AA-expressing cells than in the PEA15-DD-expressing cells (Fig. 6A, right). To test the effect of Ets-1 on IL-8 expression, we used Ets-1 siRNA to knockdown Ets-1 expression in MDA-MB-468 cells stably expressing PEA15-DD (Fig. 6B, left). Having verified Ets-1 knockdown, we examined whether Ets-1 silencing in PEA15-DD-expressing cells reduces the upregulated IL-8 levels previously observed. IL-8 mRNA expression was significantly decreased by approximately 85% upon Ets-1 knockdown (Fig. 6B, right). Furthermore, silencing of Ets-1 partly inhibited migration of cells stably overexpressing PEA15-DD (Fig. 6C). Taken together, these results suggest that PEA15 phosphorylation may regulate activity of Ets-1, leading to induction of one of its downstream targets, IL-8, thereby contributing to EMT and TNBC migration.

## Discussion

In this study, we hypothesized that PEA15 phosphorylated at both Ser104 and Ser116 is a potential contributor to the aggressiveness of TNBC. We demonstrated that expression of PEA15 unphosphorylated at both Serines had an antitumor effect in TNBC through the inhibition of EMT. Our working model postulates that suppression of migration capacity by PEA15-AA is partly due to inhibition of IL-8 expression through downregulation of the ERK downstream transcription factor Ets-1, which inhibits EMT and migration (Fig. 6D). Hence, PEA15 phosphorylation status plays a critical role in the cancer development and metastasis by determining whether PEA15 acts as either an oncogene or a tumor suppressor. Therefore, our results justify the development of phosphoinhibitory PEA15-AA as a potentially effective therapeutic molecule for the treatment of TNBC.

The roles of PEA15 in cancer development are complicated and controversial. Our group [12] and others [14, 29] reported that PEA15 binds to ERK in the cytoplasm and reduces ERK activity by downregulating transcription factor Elk-1. In colorectal cancer, Funke et al. [25] demonstrated that increased expression of PEA15 resulted in strong inhibition of proliferation and invasiveness of cancer cells. In breast cancer, Glading et al. [6] reported that low PEA15 expression is associated with aggressiveness by showing that knockdown of PEA15 expression increased the invasion of tumor cells. Similarly, previous work from our group [5] showed that PEA15 overexpression inhibited cell proliferation and induced apoptosis. Furthermore, we found that in a xenograft model, PEA15 gene therapy delivered by a adenoviral delivery system suppressed tumor growth [5, 26]. On the other hand, in other cancer types, PEA15 expression has been linked with poor prognosis and promoted metastasis [27, 28].

There is accumulating evidence showing that PEA15 mediates cellular functions depending on its phosphorylation status. For instance, Trencia et al. [29] determined that protein kinase AKT can bind and phosphorylate PEA15 on Ser116 and that its phosphorylation contributes to prevention of apoptosis. A study by Renganathan et al. [30] demonstrated that single phosphorylation at Ser104 blocks ERK binding to PEA15 and that phosphorylation at both Ser104 and Ser116 shifts the binding specificity of PEA15 from ERK to proapoptotic protein FADD, thus enhancing its anti-apoptotic function*.* Our group demonstrated that stable expression of the unphosphorylated PEA15 led to significant inhibition of ovarian tumor growth in vivo even though phosphomimetic PEA15 also showed inhibitory effects in cell proliferation, colony formation and cell migration in 2D-culture system [11, 31]. Similarly, other studies in cervical cancer and lung cancer reported that the unphosphorylated form of PEA15 serves as a tumor suppressor [32, 33].

Therefore, we examined whether modulation of PEA15 phosphorylation status at Ser104 and Ser116 could produce more potent suppression of tumorigenicity and migration than wild-type PEA15 in TNBC cells. Previously adenovirus-mediated delivery of PEA15 into mice bearing MDA-MB-468 cells caused significant tumor shrinkage and apoptosis. However, it is important to point out that wild-type PEA15 can be either monophosphorylated, double phosphorylated, or unphosphorylated in the cell; therefore, it is hard to elucidate the distinct function of PEA15 that depends on its phosphorylation status. Herein, we found that nonphosphorylatable PEA15-AA inhibited migration, and mesenchymal characteristics, which are highly related to TNBC cell properties. These data strengthen the potential for development of overexpression of unphosphorylated PEA15-AA as a TNBC-targeted therapy.

Previous studies revealed that IL-8 is involved in promoting tumor development, migration, and angiogenesis [34, 35]. Moreover, a link between IL-8, EMT, and cancer stemness has been demonstrated [36–38]. These studies supported our findings of high expression levels of IL-8 in phosphomimetic PEA15-DD-overexpressing cells. Abnormally increased ERK activity is associated with tumor progression and metastasis [39]. Jia et al. [36] reported that overexpression of IL-8 stimulated HeLa cell proliferation and migration and increased ERK activity simultaneously. PEA15 can affect ERK activity through direct binding to ERK or inhibition of phosphorylation of ERK downstream substrates such as Elk-1 and Ets-1 [40].

Our current study demonstrated that Ets-1 expression correlates with the phosphorylation status of PEA15. We found that PEA15 unphosphorylated at both Ser104 and Ser116 reversed EMT in TNBC cells by partially inhibiting IL-8 expression through Ets-1, thereby suppressing mammosphere formation and the migratory phenotype. Taken together, our results show that PEA15 phosphorylation status serves as an important regulator, having dual roles as an oncogene or tumor suppressor. Our findings provide a rationale for future development of PEA15-AA as a potential therapeutic strategy in the treatment of TNBC.

## Supplementary Information

Below is the link to the electronic supplementary material.

Supplementary file1 (DOCX 40 kb)

Supplementary file2 (PPTX 196 kb)

## Data Availability

All data generated or analyzed during this study are included in this published article.

## Abbreviations

TNBC: Triple-negative breast cancer
EMT: Epithelial-to-mesenchymal transition
PEA15: Phosphoprotein enriched in astrocytes
PEA15-AA: Nonphosphorylatable mutant form of PEA15
PEA15-DD: Phosphomimetic mutant form of PEA15
IL-8: Interleukin-8
CSC: Cancer stem cell
