# Supplementary material for: Nonphosphorylatable PEA15 mutant inhibits epithelial-mesenchymal transition in triple-negative breast cancer partly through the regulation of IL-8 expression
Source: Breast Cancer Res Treat. 2021 Jul 9;189(2):333–45. doi: 10.1007/s10549-021-06316-2 (PMC8357760; doi:10.1007/s10549-021-06316-2)
Supplement: Supplementary file 1 — Supplementary file1 (DOCX 40 kb) [file 10549_2021_6316_MOESM1_ESM.docx]

### Supplementary Methods

### Western blot analysis

Cells were washed thrice with PBS (pH 7.4; 1×) and then lysed in lysis buffer [20 mmol/L Na_2_PO_4_ (pH 7.4), 150 mmol/L NaCl, 1% Triton X-100, 1% aprotinin, 1 mmol/L phenylmethylsulfonyl fluoride, 100 mmol/L NaF, and 2 mmol/L Na_3_VO_4_] as previously described [26]. The antibodies used were rabbit anti-PEA15 polyclonal antibody (used in a 1:1,000 dilution; Cell Signaling Technology), anti-actin (1:5,000; Sigma-Aldrich), anti-fibronectin (1:500; BD Transduction Laboratories), anti-vimentin (1:1,000; Cell Signaling Technology), anti-E-cadherin (1:1,000; BD Transduction Laboratories), anti-Ets-1 (1:1,000; Cell Signaling Technology), anti-p-Ets-1 (1:1,000; Sigma-Aldrich), anti-p-STAT3 (1:1,000; Cell Signaling Technology), anti-ERK (1:1,000; Cell Signaling Technology), anti-p-ERK (1:1,000; Cell Signaling), anti-HA (1:2,500; Thermo Fisher Scientific), and anti-α-tubulin (1:5,000; Sigma-Aldrich). Secondary rabbit (1:5,000) and mouse (1:5,000) fluorescent antibodies (Molecular Probes) were detected with an Odyssey imaging system (Li-Cor Biosciences).

**Colony formation assay**

### After transfection, the three cell lines overexpressing PEA-15 mutants were harvested using trypsin and counted, and a specific number of cells (750 and 2,000 cells for MDA-MB-231 PEA15-knockout and MDA-MB-468) were plated in 6-well plates. They were cultured at 37 °C with 5% CO_2_ and saturated humidity conditions for 7-10 days. At the end, the medium was removed and cells were rinsed in PBS and fixed in 3% (v/v) acetic acid, 10% (v/v) methanol solution for 2 minutes. Then fixation solution was discarded, and 0.2% (w/v) crystal violet staining solution was added. After 30 minutes of staining, the cells were washed with water slowly and air dried, and colonies were counted manually.

**Soft agar assay**

### A bottom agarose layer (0. 75%) was placed in 12-well plates. MDA-MB-468 stable overexpressing clones were resuspended in 0.375% agarose solution in complete media at a seeding density of 10,000 cells/well. They were placed overlaid on top and incubated for 10 days. Colonies formed were stained using MTT (3-(4,5-dimethylthiazol-2-yl)-2,5-diphenyltetrazolium bromide) (Sigma), and counted those greater than 80 μm using the GelCount system (Oxford Optronix, UK) according to the manufacturer’s instructions.

**Mammosphere formation assay**

Single-cell suspensions (MDA-MB-468 cells at 2 x 10^4^ cells/well and MDA-MB-231 cells at 1 x 10^4^ cells/well) were seeded in Costar 6-well ultra-low attachment plates (Corning) using the MammoCult Human Medium Kit (StemCell Technologies). After a 7-day incubation, mammospheres were stained using MTT (3-(4,5-dimethylthiazol-2-yl)-2,5-diphenyltetrazolium bromide) (Sigma), and spheres greater than 80 μm in diameter were counted using the GelCount system (Oxford Optronix) according to the manufacturer’s instructions.

**Transwell migration assay**

As described previously [27], migration assays were performed in triplicate using a 24-well micro-chemotaxis chamber. Cells (1 x 10^5^/350 μL) were resuspended in FBS-free medium and added into each chamber. The bottom chamber was filled with complete medium (750 μL) containing 10% FBS as an attractant. The cells were allowed to migrate for 6-24 hours and then were fixed and stained with hematoxylin and eosin. For investigating IL-8 and neutralizing antibody, the bottom chamber contained 0.5% FBS containing IgG isotype control (25μg/mL), recombinant human IL-8 (500ng/mL, Invitrogen), or purified NA/LE Mouses anti-Human IL-8 (25μg/mL, BD Biosciences) and allowed to migrated for 24 hours. Migrated cells were scanned using the PathScan Enabler IV histology slide scanner (Meyer Instruments) and then quantified using National Institutes of Health ImageJ software (http://rsb.info.nih.gov/ij/).

### Immunohistochemistry

As described previously [27], tumor tissues were fixed in neutral-buffered formalin and embedded in paraffin. Sections (5 µm each) were prepared using a microtome, mounted on slides, deparaffinized in xylene, rehydrated in graded alcohols, and washed in distilled water. Antigens were retrieved by boiling the sections in 10 mM citric acid (pH 6.0) for 40 minutes. Endogenous peroxidases were quenched by incubation in 3% H_2_O_2_ for 10 minutes at room temperature. The slides were washed three times with PBS and blocked for 30 minutes with 10% normal goat serum in 1% bovine serum albumin/PBS. The slides were then exposed to the following antibodies: anti-Ki-67 (Lab Vision), anti-vimentin (1:100; Cell Signaling), anti-E-cadherin (1:250; BD Transduction Laboratories), and anti-HA (1:500; Covance). Stained slides were visualized with an Eclipse 80i microscope (Nikon Instruments Inc). Intensity of immunohistochemical staining of target proteins was measured using the Vectra 3 automated quantitative pathology imaging system (PerkinElmer Inc).

**Protein expression and purification**

The gene coding for PEA15-AA fused with rEgH9 and TDP (rEgH9-TDP-PEA15-AA) was cloned into pET21a vector (Novagen), and a hexa-histidine tag was also introduced to the C-terminus of the corresponding genes for affinity purification. Origami B (Merck Bioscience) cells were transformed with the constructed vector. The protein was over-expressed by addition of 0.5 mM of isopropyl-d-1-thiogalactopyranosid (IPTG) when OD_600_ reached about 0.6. After 20 hr, the cells were disrupted by sonication, and the protein was purified through affinity and gel permeation chromatography (Superdex200, GE Healthcare) with a phosphate-buffered saline (PBS; pH 7.4). The protein concentration was determined using UV–Vis spectrophotometry at 280 nm.

**Small interfering RNA and electroporation**

IL-8 siRNA, Ets-1 siRNA, and negative control siRNA (ON-TARGETplus Non-targeting Pool) were purchased from Sigma-Aldrich. MDA-MB-468 cells were trypsinized and resuspended at 1 × 10⁷ cells/mL in Resuspension Buffer R (Thermo Fisher Scientific). We mixed 100 μL (1 × 10^6^ cells) of this cell suspension with siRNA (100 nM) added to a Neon Tip (Thermo Fisher Scientific). We electroporated (25 ms, 1100V) 1 × 10^6^ cells with 100 nM of siRNA in 100 μL of medium (Thermo Fisher Scientific). Cells were then resuspended in complete DMEM, incubated for 48 hours, and used for further experiments.

### Quantitative RT-PCR

Total RNA was purified from cells using an RNA prep kit (Invitrogen) according to the manufacturer’s instructions. Next, 20 ng of total RNA was used for one-step quantitative reverse transcription PCR (RT-qPCR) analysis with the iScript One-Step RT-PCR kit including SYBR Green reagents using a CFX96 real-time PCR detection system (Bio-Rad Laboratories). PCR conditions were 94°C for 5 minutes followed by 40 cycles of 94°C for 15 seconds and 60°C for 30 seconds. Human Snail, vimentin, Slug, N-cadherin, fibronectin, Zeb1, Twist, IL-8, Ets-1, and GAPDH RNA primers (Table 1) were purchased from Sigma-Aldrich. GAPDH levels were used as an endogenous control. The real-time PCR data were calculated using the comparative threshold cycle method and the iCycler CFX96 analyzer software (Bio-Rad).

**Table1.**

Quantitative reverse transcription PCR primer sequences
