# Supplementary figures and images for: Nonphosphorylatable PEA15 mutant inhibits epithelial-mesenchymal transition in triple-negative breast cancer partly through the regulation of IL-8 expression
Source: Breast Cancer Res Treat. 2021 Jul 9;189(2):333–45. doi: 10.1007/s10549-021-06316-2 (PMC8357760; doi:10.1007/s10549-021-06316-2)

## Slide 1
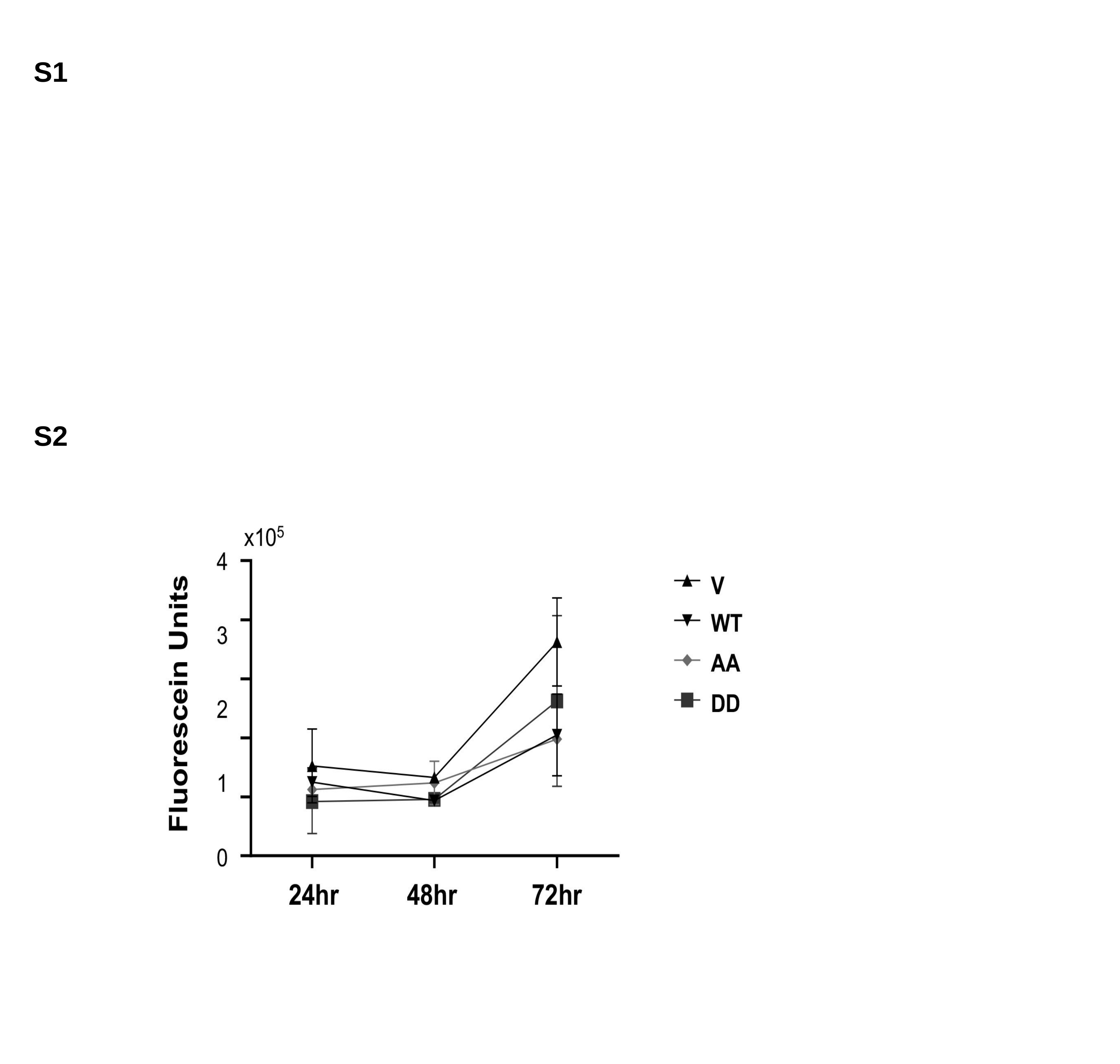

S1
S2

Supplement: Supplementary file 2 — Supplementary file2 (PPTX 196 kb) [file 10549_2021_6316_MOESM2_ESM.pptx]
